# Drinking and Flying: Does Alcohol Consumption Affect the Flight and Echolocation Performance of Phyllostomid Bats?

**DOI:** 10.1371/journal.pone.0008993

**Published:** 2010-02-01

**Authors:** Dara N. Orbach, Nina Veselka, Yvonne Dzal, Louis Lazure, M. Brock Fenton

**Affiliations:** 1 Department of Biology, University of Western Ontario, London, Ontario, Canada; 2 Department of Biology, University of Regina, Regina, Saskatchewan, Canada; University of Oxford, United Kingdom

## Abstract

**Background:**

In the wild, frugivorous and nectarivorous bats often eat fermenting fruits and nectar, and thus may consume levels of ethanol that could induce inebriation. To understand if consumption of ethanol by bats alters their access to food and general survival requires examination of behavioural responses to its ingestion, as well as assessment of interspecific variation in those responses. We predicted that bats fed ethanol would show impaired flight and echolocation behaviour compared to bats fed control sugar water, and that there would be behavioural differences among species.

**Methodology/Principal Findings:**

We fed wild caught *Artibeus jamaicensis, A. lituratus, A. phaeotis, Carollia sowelli, Glossophaga soricina*, and *Sturnira lilium* (Chiroptera, Phyllostomidae) sugar water (44 g of table sugar in 500 ml of water) or sugar water with ethanol before challenging them to fly through an obstacle course while we simultaneously recorded their echolocation calls. We used bat saliva, a non-invasive proxy, to measure blood ethanol concentrations ranging from 0 to >0.3% immediately before flight trials. Flight performance and echolocation behaviour were not significantly affected by consumption of ethanol, but species differed in their blood alcohol concentrations after consuming it.

**Conclusions/Significance:**

The bats we studied display a tolerance for ethanol that could have ramifications for the adaptive radiation of frugivorous and nectarivorous bats by allowing them to use ephemeral food resources over a wide span of time. By sampling across phyllostomid genera, we show that patterns of apparent ethanol tolerance in New World bats are broad, and thus may have been an important early step in the evolution of frugivory and nectarivory in these animals.

## Introduction

As fleshy fruits ripen, their sugars are converted to ethanol by *in situ* fermentation, mediated by microorganisms [1]–[3]. Ripe fruits generally have higher concentrations of ethanol than unripe ones [3]–[4]. Although studies on ethanol concentrations of fruits in natural ecosystems are limited [4]–[5], available information suggests that in Central America, ethanol concentrations in fermenting fruits can range from 0.6% up to 4.5% [3]. Thus frugivores and nectarivores in the tropics may be exposed to fruits with a wide range of concentrations of ethanol and, while foraging, may naturally encounter it in high concentrations [6]. Frugivorous animals display diverse responses to consuming ethanol (reviewed in [3]–[5]), and where an individual falls on the spectrum of tolerance could influence its ability to exploit certain foods.

The effects of ethanol consumption can be negligible or beneficial for some frugivores (reviewed in [3]–[4]). Pentail treeshrews (*Ptilocercus lowii*) feeding on nectar of bertam palms (*Eugeissona tristis*) often consume ethanol in doses exceeding 1.4 g ethanol /kg body mass (enough to legally intoxicate humans) without demonstrating signs of intoxication or deleterious effects [6]. Conversely, several fruit-eating animals (e.g., cedar waxwings, *Bombycilla cerdorum*) are adversely affected when they eat large quantities of fruit rich in ethanol [7]–[11]. In humans and some other primates, elevated blood ethanol concentrations (BACs) can affect performance, impairing reaction times, manoeuvrability, spatial orientation, and reasoning [12]–[14], which may lead to reduced survival. Frugivorous bats use odour [15], and sometimes echolocation cues [16]–[17], to locate fruits or assess ripeness [18]–[22]. Many frugivorous and nectarivorous animals find overripe fruit unpalatable and may avoid inebriation by consuming fruit with low ethanol content, or consuming smaller quantities of alcohol-rich food [7], [18], [23].

The effects of ethanol are not entirely deleterious. Ethanol can help stimulate appetite and increase net energy intake in humans [24]–[26]. Ethanol plumes emitted by ripe fruits provide olfactory cues that may potentially increase animals' foraging success [2], [27]. Given that ethanol has a high energy content, exposure to it should select for processes that aid in its metabolism while minimizing costs such as motor impairment [3]. After consuming ethanol, honeybees (*Apis mellifera*) increase flower visitation rates but show no signs of motor impairment [28]. While trade-offs between the costs of consuming toxic foods and the energy rewards has received some attention [29]–[30], previous studies on ethanol ingestion by frugivorous bats have used captive animals repeatedly exposed to ethanol [18], [29], [31]–[32], and no published studies have examined the flight-motor impairment of bats consuming large amounts (≥1%) of ethanol. By examining behavioural responses of wild bats that may have built up natural tolerances for ethanol, we hope to expand the ecological scope of intoxication studies.

We predicted that consumption of ethanol by bats would lead to levels of intoxication (∼0.11 g.100 ml^−1^ blood alcohol content) that would impair both flight performance (motor control) and echolocation (orientation). Specifically, we predicted that bats fed ethanol would require more time to manoeuvre through an obstacle course, collide with more obstacles, land to rest more often, and not complete the course as often as our control group [33]. If ethanol impairs echolocation, then its consumption may alter features of bats' echolocation calls such as pulse durations, interpulse intervals and frequency characteristics of calls compared to those of control individuals [34].

## Methods

### Ethics Statement

Our study was conducted from 21–28 April 2009 at Lamanai Outpost Lodge, Belize (17°45′N; 88°39′W) using 3 *Artibeus jamaicensis,* 7 *A. literatus,* 35 *A. phaeotis,* 19 *Carollia sowelli,* 11 *Glossophaga soricina* and 31 *Sturnira lilium* (Chiroptera, Phyllostomidae). The bats were wild caught and none of the females was visibly pregnant or lactating. We captured and immediately removed bats from mist nets between dusk and midnight along roadways, forest trails, and small clearings within 2 km of the Lodge. During feeding treatments, bats showed no signs of pain or discomfort. We held bats fed ethanol for one hour after their flight trials so that the treatment wore off or lessened before we released them at the site of capture. This research was conducted under permit No. 4/1981 of the Belizean Scientific Collection/Research Permit Wildlife Protection Act, and protocol No. 2008–003 of the University of Western Ontario's University Council on Animal Care.

### Obstacle Course

**Figure 1:**
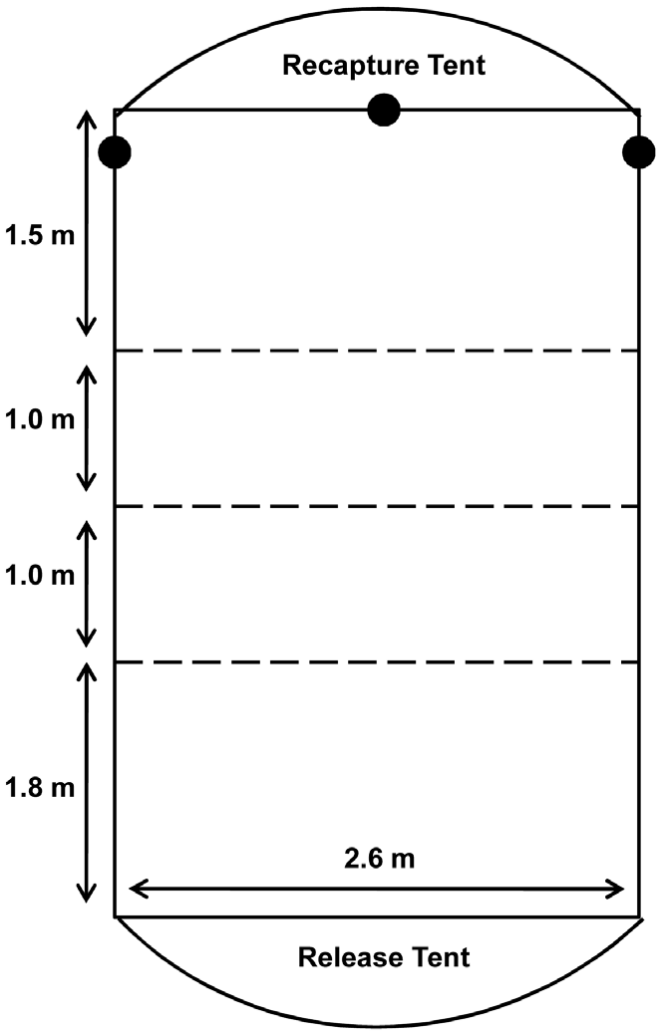
Flight corridor and obstacle spacing. The flight corridor used for each trial. Broken lines represent rows of obstacles, and black circles represent microphones.

We constructed a wooden flight corridor (5.3 m×2.6 m×1.5 m), lined with clear polyethylene plastic to prevent bats from roosting or escaping (Fig. 1). To test manoeuvrability and flight behaviour, we secured obstacles (plastic linked chains, 1.2–1.5 m long, and 0.025 m in diameter) in three parallel off-centered rows spaced 1 m apart. We adjusted the spacing of obstacles within a row to be approximately one wingspan estimated according to each bat's forearm length, using a linear regression based on measurements of forearm lengths and wingspans [35–36; Table 1] of several Belizean bat species [37], where Wingspan (in cm)  = 0.68 × (Forearm length – in mm). The obstacle course was designed to challenge each bat's manoeuvrability rather than their aerodynamic limits. Distances between obstacles (one wingspan) represented a very cluttered environment [38], and this approach has been used successfully in other studies of bat flight performance [39].

**Table 1:** Morphological measurements.

Species	N	Body mass (g)	Forearm length (mm)	Wingspan (cm)
*Artibeus literatus*	7	57.74±9.40	69.62±4.10	47.14±2.78
*Artibeus jamaicensis*	3	47.80±5.43	61.91±1.16	41.92±0.78
*Artibeus phaeotis*	35	12.21±1.36	38.82±2.51	26.28±1.70
*Carollia sowelli*	19	16.24±2.21	40.35±1.90	27.32±1.29
*Glossophaga soricina*	11	9.98±0.95	35.40±1.76	23.97±1.19
*Sturnira lilium*	31	16.06±1.78	38.15±3.75	25.83±2.54

Morphological measurements of bats used to calculate obstacle spacing and feeding volumes. Body mass, forearm length, and wingspan (derived from 0.68 times forearm length) measurements are 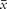 ± S.D. n  =  sample size.

### Feeding Treatments

Bats were fed either a sugar water control (44 g of table sugar in 500 ml of water), or 1.5% ethanol with sugar water (40% ethanol diluted in sugar water with the same concentration of sugar as the control). During preliminary trials, we determined that our sugar concentration stimulated bats to drink rapidly, and willingness to feed appeared to depend on the individual bat and not the feeding treatment. The feeding treatment was randomly selected for each bat, which only received one treatment.

To our knowledge, there is no information on alcohol absorption and metabolism kinetics in phyllostomid bats. Work on the consumption of ethanol-rich foods by Old World fruit bats (*Pteropodidae*) has assumed ethanol kinetics similar to those of humans [32] so we administered ethanol in proportion to total blood volume [40]. Thus, a 16 g bat should require 1.03 ml of ethanol sugar water to achieve a BAC of 0.11 g 100 ml^−1^ [32], [41], which we expected would produce overt effects of intoxication. We weighed each bat and orally fed it enough sugar water with ethanol to attain a BAC of 0.11 g 100 ml^−1^, or the equivalent volume of ethanol-free sugar water as a control. Ethanol concentrations in saliva are comparable to those in blood samples measured simultaneously [42]–[44]. We evaluated BACs in bats using ethanol-sensitive reagent pads placed in the mouth for 30 s (ALCO-Screen®, Chematics, USA). These pads react to ethanol in saliva and produce a colour corresponding to 0%, 0.02%, 0.04%, 0.08% or >0.3% BAC [45]. When blood-alcohol concentrations ≥0.10%, the ALCO-Screen test is 90% sensitive and 92% efficient [44]. When tested with the ALCO-Screen reagent pads, most of the treatment bats showed evidence of ethanol in their saliva. Previous work using ALCO-Screen reagent pads generally indicate ethanol concentrations lower than those in the blood [43], so the BACs we report for individual bats may underestimate their actual BACs. Because we compared ethanol concentrations in saliva only, all measurements are relative and the resulting comparisons are appropriate for discussing the implications of ethanol consumption. Although ethanol passively diffuses into the saliva from blood plasma, ethanol may remain in the mouth after ingestion and this could contaminate BAC readings when saliva is tested immediately after alcohol ingestion [46]. Therefore we waited 15 minutes between feeding a bat and taking its BAC reading. Although alcohol absorption rates are highly variable in humans, alcohol concentrations generally peak in the blood stream 30 to 60 min following consumption on an empty stomach due to the accelerated rate of gastric emptying and subsequent absorption in the small intestine [47]. Since we assumed the same pharmacokinetics of alcohol absorption in bats as in humans, testing BAC using alcohol test strips 15 min after alcohol ingestion allowed us sufficient time to run the test and transport the animals to the flight cage before the effects of the ethanol began to wear off.

### Flight Performance

Each trial consisted of one bat flying once through three rows of obstacles. We videoed its flight performance using a night-sensitive camera (model DCR-SR65, Sony) and infrared lights (model IRLamp6, Wildlife Engineering). If a bat did not fly immediately, we induced flight by approaching it and snapping our fingers. We measured five flight characteristics: 1) time spent in flight during the trial; 2) course completed or not completed (flying from the release tent to the recapture tent); 3) circling behaviour exhibited or not exhibited (flying in a circle); 4) landing on flight corridor supports/obstacle chains or no landing; and 5) whether or not the bat flew back to the point of release. Bats never collided with obstacles, irrespective of the treatment. Therefore, we did not include collision data in our analysis. We analyzed videos using MotionDV Studios (Panasonic version 5.3E, Matsushita Electric Industrial Co., Japan).

### Echolocation

We used an array of four ultrasonic condenser microphones (Avisoft UltraSoundGate 416, Avisoft Bioacoustics, Germany) to record each bat's echolocation calls as it negotiated the obstacle course. Recordings were digitized at a sampling rate of 250 kHz and a resolution of 8 bits, and stored as wav-files with Avisoft Recorder USG software (v.2.9. Avisoft Bioacoustics, Germany). We affixed three microphones to the corridor supports at the terminal end of the corridor, facing the oncoming bat. One microphone was 1.2 m high and centred in the middle of the corridor, while two microphones were positioned 1 m above the ground on each side. A fourth microphone was affixed to the camera tripod and pointed toward one experimenter who marked the beginning and end of each bat's flight on the acoustic files with an ultrasonic whistle (Dog Dazer II, Kii Enterprises, USA). We analyzed sequences of eight consecutive echolocation calls from the terminal end of each bat's flight. Files with calls below average background noise levels or less than 30 KHz were excluded. Using Pulse Characteristics Analysis in BatSound Pro (v.3.31b, Petterson Electronik AB, Sweden), we measured three call variables: frequency of most energy (FME), interpulse interval (IPI), and pulse duration (PD).

### Statistics

We had small sample sizes for *A. jamaicensis* (n = 3) and *A. literatus* (n = 7), but because they are the same genus and physically bigger than the other species (Table 1), we pooled the data for them for use in our analysis. We used analysis of variance (ANOVA) followed by a Tukey's HSD *post-hoc* test (SPSS v.16, SPSS Inc., USA) to determine the effects of treatments and species (*A. jamaicensis/literatus, A. phaeotis, C. sowelli, G. soricina*, or *S. lilium*) on time spent flying during trials. We used binary logistic regressions, with species and treatments as predictors, to test four additional flight behaviours: course completion, circling, landing, and returning to release point. We set *A. jamaicensis/literatus* as the comparison group for flight behaviours to test for a size effect. We used multivariate analysis of variance (MANOVA) followed by Tukey's HSD *post-hoc* tests to determine the effects of treatments and species on echolocation variables (FME, IPI, and PD). We log_10_ transformed time spent flying and PD data to meet assumptions of normality. Non-significant interaction terms were removed from the models. We used a Fisher's exact test to compare the blood alcohol concentrations of bats fed alcohol between species.

## Results

### Flight Performance

**Figure 2:**
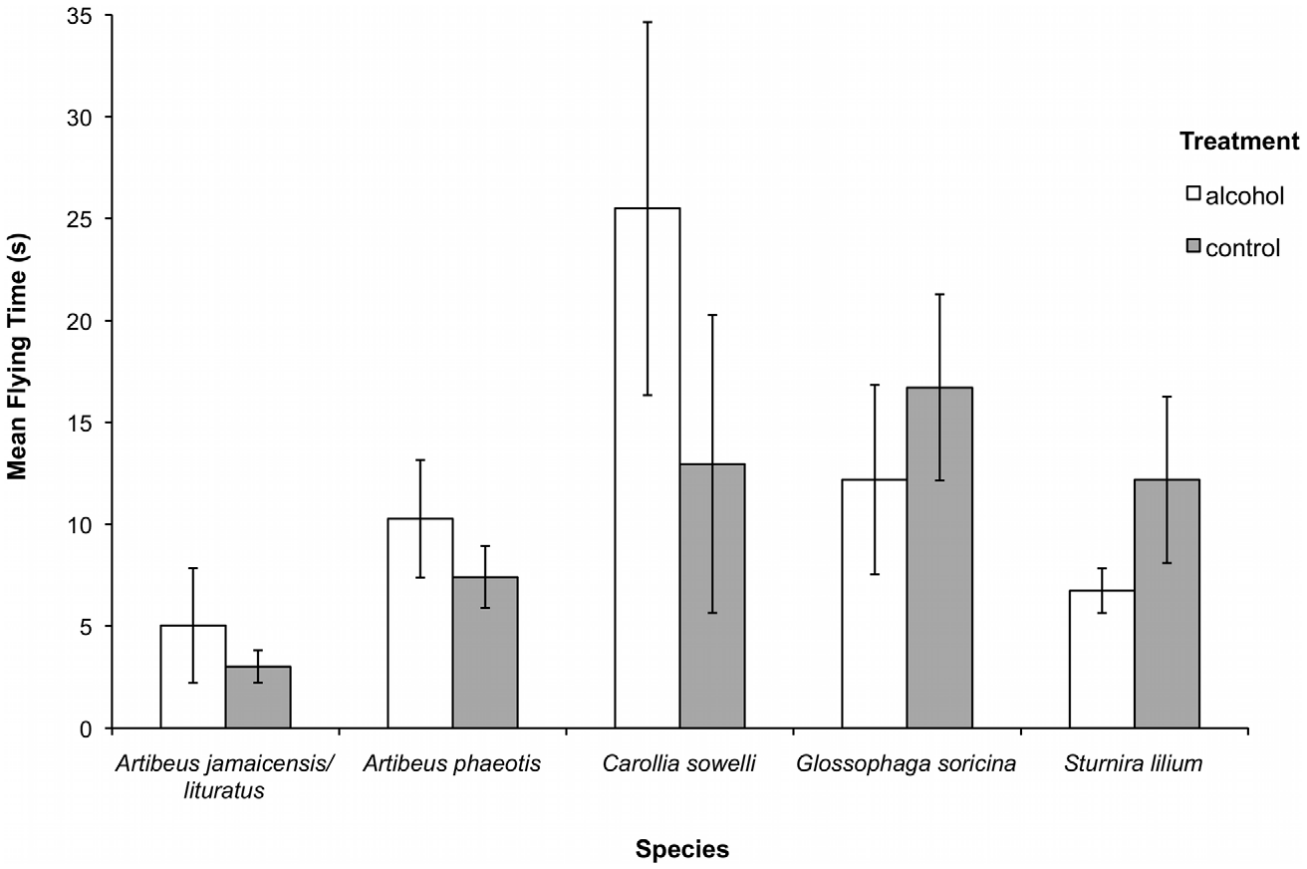
Mean flight times through obstacle course. Comparison of mean flight times (sec ± SE) through an obstacle course by phyllostomids fed 1.5% alcohol and those fed a sugar water control.

Flying time through the obstacle course differed among species (ANOVA, F_4, 73_ = 3.217, *p* = 0.017), but not between treatments (ANOVA, F_1, 73_ = 0.115, *p* = 0.736; Fig. 2). *Carollia sowelli* (Tukey's, 20.5±19.8 s, mean ± SD) and *G. soricina* (14.4±10.0 s) took significantly longer to complete a trial than *A. jamaicensis/literatus* (3.9±3.2 s). Course completion differed significantly between *A. phaeotis* and *A. jamaicensis/literatus* (Wald_1_ = 4.18, *p* = 0.041), with *A. phaeotis* completing 94% of trials, while *A. jamaicensis/literatus* completed only 70% of trials. Treatment (exposure to ethanol) had no effect of on course completion (Wald_1_ = 1.72, *p* = 0.189). There was a significant difference between *S. lilium* and *A. jamaicensis/literatus* on whether or not bats returned to the point of release (Wald_1_ = 3.87, *p* = 0.049). *Sturnira lilium* flew back to the point of release in 58% of the trials, whereas *A. jamaicensis/literatus* flew back in only 20% of the trials. Treatment had no effect on returning to the point of release (Wald_1_ = 0.11, *p* = 0.738). Circling behaviour (species: *p>*0.05; treatment: Wald_1_ = 1.01, *p* = 0.314) and landing behaviour (species: *p>*0.05; treatment: Wald_1_ = 0.04, *p* = 0.838) did not differ significantly among species or between treatments.

### Echolocation

Treatment had no effect on echolocation call characteristics (MANOVA, F_3, 84_ = 1.17, *p* = 0.325), but there were differences among species (MANOVA, F_12, 223_ = 11.55, *p*<0.001) in frequency with most energy (F_4, 86_ = 28.05, *p*<0.001), interpulse interval (F_4, 86_ = 4.45, *p* = 0.003) and pulse duration (F_4, 86_ = 7.57, *p*<0.001). Tukey's *post-hoc* tests revealed that the frequency with most energy was lowest in *A. jamaicensis/literatus* (71.5±8.0 kHz, mean ± SD) followed by *C. sowelli* (81.8±8.3 kHz), *S. lilium* (90.3±8.3 kHz), and *A. phaeotis* (98.1±8.0 kHz), respectively, and was highest in *G. soricina* (108.5±12.1 kHz). Additionally, *A. jamaicensis/literatus* produced longer calls (Tukey's, 9.48×10^−1^±2.16×10^−1^ ms, mean ± SD) than *C. sowelli* (4.45×10^−1^±2.29×10^−1^ ms), *A. phaeotis* (4.57×10^−1^±2.09×10^−1^ ms), and *G. soricina* (4.73×10^−1^±2.47×10^−1^ ms).

### BAC

**Figure 3:**
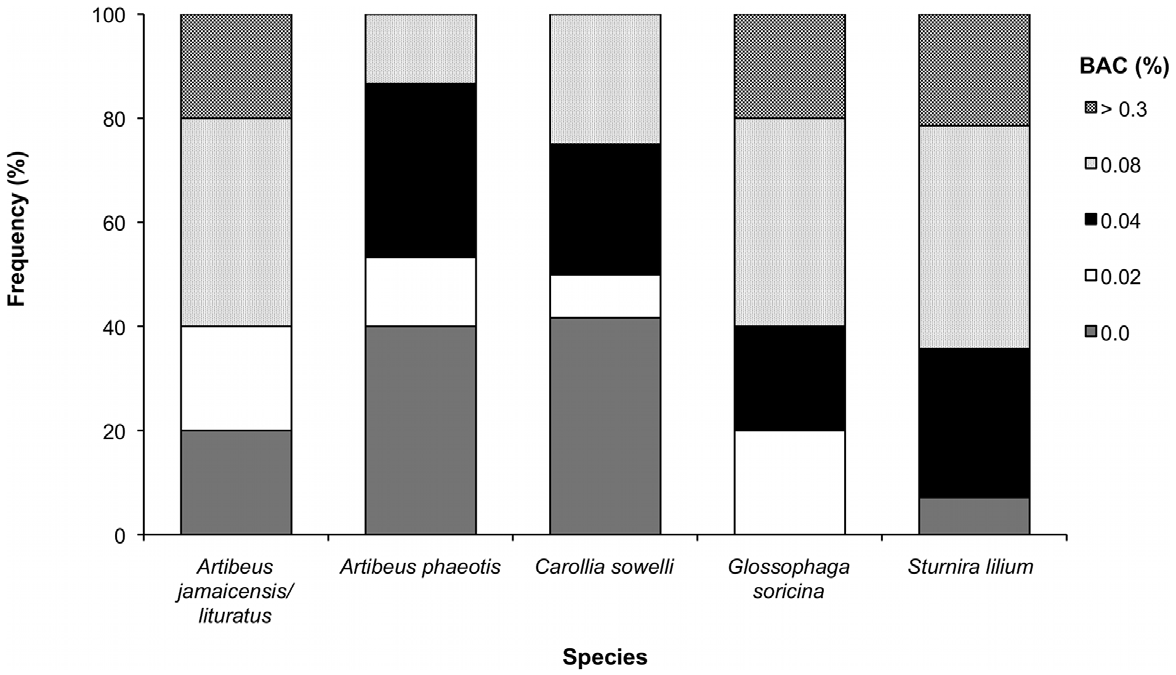
Blood alcohol concentration histograms. Frequency histograms of blood alcohol concentrations for *Artibeus jamaicensis/literatus* (n = 5), *Artibeus phaeotis* (n = 15), *Carollia sowelli* (n = 12), *Glossophaga soricina* (n = 5), and *Sturnira lilium* (n = 14) fed 1.5% alcohol.

There was a significant difference in blood alcohol concentrations among species (Fisher's exact test, p<0.001), with *A. jamaicensis/literatus, G. soricina,* and *S. lilium* appearing to have higher BACs than *A. phaeotis*, and *C. sowelli* (Fig. 3).

## Discussion

We found no significant differences in flight performance or echolocation behaviour between bats fed sugar water and bats fed ethanol, suggesting that wild frugivorous and nectarivorous phyllostomids tolerate ethanol at the concentration we studied. Naturally fermenting fruits can range from 0.6% up to 4.5% ethanol in Central America [3]. The 1.5% ethanol concentration we fed bats does not appear to have behavioural repercussions, and may be encountered regularly by foraging bats.

In humans and rodents, genetic factors may explain tolerance to ethanol [48]–[49], Variation in expression of alcohol dehydrogenase and aldehyde dehydrogenase, the primary enzymes involved in the breakdown of alcohol, is a good predictor of individual tolerance of ethanol. Environmental factors, such as long-term dietary exposure to ethanol, may also be involved [50]. By examining wild caught bats' behavioural responses to ethanol, we apply ecologically relevant interpretations, and examine the possibility of susceptibility to environmental influence. This avoids confounding artefacts manifest in captive studies, such as training periods and development of unnatural ethanol tolerance from repeated exposures.

Orbach (unpublished observations from July 2008) observed that flying captive *Rousettus aegyptiacus* (Pteropodidae) were less able to successfully negotiate an obstacle course (obstacles 1 wingspan apart) after consuming fruit juice spiked with ethanol than bats just fed fruit juice. Fleshy fruits in the Neotropics may ferment faster and have higher ethanol concentrations than those in Mediterranean habitats, possibly reflecting differences in humidity, fruit abundance, and prevailing temperatures [51]–[52] **.** Thus foraging Neotropical fruit- and nectar-feeding bats may naturally be exposed more often to comparatively high levels of ethanol (up to 4.5% [3]) compared to those in Mediterranean areas (up to 0.7% [31]), and therefore may have higher tolerances for ethanol. However, more research determining naturally occurring concentrations of ethanol in fruits vulnerable to fermentation are necessary to validate this hypothesis.

Feeding states may also be important in determining a bat's likelihood to consume foods rich in ethanol. *R. aegyptiacus* frequently eat fruits containing 0.1–0.7% ethanol concentrations [31], and during food-choice experiments, avoid foods with >1% ethanol [18], [29], [31]. However, food-deprived *R. aegyptiacus* do not discriminate against foods based on ethanol content and eat foods with high (≥1%) concentrations of ethanol [29], presumably to compensate for dietary energy shortages, as has been proposed for food-deprived hamsters and mice [53]–[54] **.** When availability of palatable fruit is limited by seasonality or weather, some frugivorous bats supplement their diet with insects, pollen, nectar, flowers, or leaves [55]–[59]. As had been proposed for *R. aegyptiacus* [29], we expect that frugivorous phyllostomids may consume more fruits rich in ethanol more often when other food is unavailable.

Food-deprived rats have slowed ethanol metabolism rates compared to controls [60] and the same may be true of food-deprived bats. This would mean longer effects of ethanol compared to well-nourished bats or rats. In places like Israel, where ripe fruit availability is seasonal, the possibility of food-restriction is real [57], and bats may be susceptible to predation risks and intoxication. The wild phyllostomids we studied had been captured while foraging and may have had residual food in their stomachs to help absorb ethanol and mitigate its impact [61]. Future studies using food-deprived phyllostomids or increased concentrations of ethanol may reveal greater behavioural impact of ethanol that could be important to understanding survival probabilities.

Interspecific variation we observed in flying time, course completion, and return to the release point, irrespective of feeding treatments, may be part of a suite of interspecific dietary [62]–[63] and habitat differences [35] among phyllostomids. Body mass does not appear to affect flight performance, although it may account for changes in echolocation behaviour. Differences among species in echolocation call characteristics could reflect morphological variation, as the call frequencies of bats increase as their body mass decreases [(Table 1); 64].

Our bats appeared to be behaviourally unaffected by ethanol at the concentration we used, although we observed interspecific variation in blood alcohol concentrations. By sampling widely across phyllostomid species of different masses, we demonstrate that the patterns of exposure to, and tolerance of ethanol are broad, and suggest that sensitivity to ethanol may be important in the adaptive radiation of frugivorous and nectarivorous bats. Across the tropics there is variation in the diversity of frugivorous bats, ranging from 25 genera in the Neotropics to 11 in the Ethiopian tropics to 21 in the East Indies [65]. The properties of fruits, such as their temporal and spatial distribution, size, and resistance to mechanical deformation, may contribute to this diversity within regions [reviewed in 66]. For example, variation in biting styles and dentition may allow some bats to exploit harder fruits inaccessible to sympatric species [59]. Future long-term studies contrasting the tolerance of fruit- and insect-feeding phyllostomids to ethanol may provide another factor explaining the diversity of New World fruit-feeding bats, and confirm the role of alcohol tolerance in the evolution of frugivory. We advise using alternative methods to assess the BACs of bats other than alcohol-sensitive reagent pads due to the highly subjective nature of interpreting colour blots. If tropical and subtropical fruits contain higher levels of alcohol more often than those in temperate environments (e.g., Mediterranean climates), ethanol tolerance of other frugivores and nectarivores elsewhere in the tropics and subtropics may indicate parallels among Old World fruit-feeding bats. Furthermore, species like *R. aegyptiacus*, which occur widely from South Africa to Turkey, may show geographic variation in ethanol tolerance reflecting a range that brackets tropical and subtropical climates.
